# Accumulation of Small-Size, Highly Dispersive Mesoporous Silica Nanoparticles in a Tumor in Both Chorioallantoic Membrane and Mouse Models

**DOI:** 10.3390/cells14100734

**Published:** 2025-05-17

**Authors:** Aoi Komatsu, Yuya Higashi, Cong-Kai Lin, Yi-Ping Chen, Si-Han Wu, Minoru Suzuki, Kotaro Matsumoto, Fuyuhiko Tamanoi

**Affiliations:** 1 Institute for Integrated Cell-Material Sciences, Institute for Advanced Study, Kyoto University, Kyoto 606-8501, Japan; komatsu.aoi.6z@kyoto-u.ac.jp (A.K.); higashi.yuya.5n@kyoto-u.ac.jp (Y.H.); 2 Graduate Institute of Nanomedicine and Medical Engineering, Taipei Medical University, Taipei 11031, Taiwan; qrq123456789@gmail.com (C.-K.L.); haychen@tmu.edu.tw (Y.-P.C.); smilehanwu@tmu.edu.tw (S.-H.W.); 3 International Ph.D. Program in Biomedical Engineering, Taipei Medical University, Taipei 11031, Taiwan; 4 Institute for Integrated Radiation and Nuclear Science, Kyoto University, Osaka 590-0494, Japan; suzuki.minoru.3x@kyoto-u.ac.jp

**Keywords:** CAM model, patient-derived cells, osteosarcoma, mesoporous silica nanoparticles, tumor accumulation

## Abstract

(1) Background: The chorioallantoic membrane (CAM) model has the potential to contribute to the development of personalized medicine based on individual cancer patients. We previously established the CAM model using patient-derived *CIC-DUX4* sarcoma cells. We also used the CAM model for characterization and a comparison with the mouse model by examining the tumor accumulation of small-size, highly dispersive mesoporous silica nanoparticles (MSNs). (2) Method: In this study, we transplanted a variety of cancer cell lines, including patient-derived osteosarcoma (OS) and extraskeletal osteosarcoma (ESOS) cells. Patient-derived OS, ESOS and other cell lines were transplanted onto CAMs. The proliferation of cancer cells within CAM tumors was confirmed using H&E staining. For the comparison of the CAM and mouse models, rhodamine B-labeled MSNs were administered intravenously to CAMs and to xenograft mice. Tumor accumulation was evaluated by examining fluorescence and by confocal microscopy. The biodistribution of MSNs was examined by measuring the Si content by ICP. (3) Results: H&E staining demonstrated the proliferation of cancer cells of OS, ESOS and others on CAMs. While growth patterns and morphologies varied among different cancer types, H&E staining confirmed the establishment of tumors. As for the tumor accumulation, both the CAM and mouse models showed that MSNs were selectively accumulated in the tumors in both the CAM and mouse models. (4) Conclusions: We have expanded the range of CAM models by using a variety of cancer cells, including patient-derived cell lines. We also report that the small-size, highly dispersive MSNs exhibit excellent tumor accumulation in both the CAM and mouse models. These results point to the usefulness of the CAM model for patient-derived cancer cells as well as for evaluating drug carriers for tumor targeting.

## 1. Introduction

Cancer is highly heterogeneous, and patients exhibit different genetic mutation profiles and drug responses even with the same cancer type [1,2]. To understand this tumor diversity and facilitate the development of individual treatments, animal models that accurately reflect patient tumor characteristics are essential. While mouse models have been widely used [1,2], we, as well as others, have developed the chorioallantoic membrane (CAM) assay [3,4,5,6]. This model utilizes the chorioallantoic membrane of fertilized chicken eggs, enabling tumor formation in approximately 5 days after transplantation of cancer cells. This rapid tumor formation is attributed to the nutrient-rich environment of the CAM and the incomplete establishment of the immune system in the chicken embryo [7,8]. We previously reported the establishment of a patient-derived chicken egg model (PDcE) [9,10]. This model has significant potential to contribute to the development of personalized medicine tailored to individual cancer patients in the future. To this end, we previously demonstrated the utility of the CAM model using patient-derived *CIC-DUX4* sarcoma [10]. We verified that the transplanted CAM tumors retained both the *CIC-DUX4* fusion gene and the characteristic features of the original patient tumor. In the current study, we further expand the CAM model by using various cancer cells, including two types of patient-derived osteosarcomas, typical osteosarcoma (OS) and extraskeletal osteosarcoma (ESOS) [11,12]. OS is one of the most common malignant bone tumors, primarily arising in bones and frequently occurring in adolescents [13]. It exhibits highly aggressive behavior with poor prognosis in cases presenting distant metastasis at diagnosis or showing inadequate response to adjuvant chemotherapy [14,15]. ESOS, a rare mesenchymal malignancy, develops in soft tissues independent of bone or periosteum. While OS predominantly affects children and adolescents, ESOS shows a higher incidence in adults, particularly males aged over 50 years [16,17]. Both osteosarcomas share comparable chemotherapy protocols, although the efficacy of these regimens remains limited, particularly in ESOS [18]. Metastatic ESOS cases demonstrate especially poor outcomes with 5-year survival rates as low as 10% [19]. Classified as rare cancers (<6 cases/100,000 population), their low incidence complicates mechanistic studies, as well as the development of effective treatment strategies [20,21,22].

In this paper, we also report on our study performed to characterize tumor accumulation of small-size, highly dispersive mesoporous silica nanoparticles (sdMSNs) [23,24] in the CAM and mouse models. These nanoparticles have emerged as a promising type of drug carrier due to their remarkable tumor accumulation in a mouse model. Because MSNs harbor a large surface area, tunable particle and pore size and high biocompatibility [25,26,27], these sdMSNs are a promising drug carrier for cancer therapy. We first confirmed and extended their tumor accumulation capability in a mouse model. We then characterized tumor accumulation in the CAM model. Our results demonstrate that sdMSNs show similar tumor accumulation in both the CAM and mouse models. Taken together, these results point to the idea that the CAM model provides a simple and versatile cancer model.

## 2. Materials and Methods

### 2.1. Chemicals

Rhodamine B isothiocyanate (RBITC) was purchased from Sigma-Aldrich (St. Louis, MO, USA). Cetyltrimethylammonium bromide (CTAB), tetraethyl orthosilicate (TEOS) were purchased from Acros Organics (Geel, Belgium). 2-[Methoxy(polyethyleneoxy)6-9propyl] trimethoxysilane (PEG-silane) and N-trimethoxysilylpropyl-N,N,N-trimethylammonium chloride 50% in methanol (TA-silane) were obtained from Gelest (Morrisville, PA, USA). Hoechst33258 (Hoechst) was purchased from Dojindo Laboratories (Kumamoto, Japan).

### 2.2. Preparation and Characterization of MSN-PEG/TA

Small-size, highly dispersive MSNs were prepared as described by Chen et al. [24]. Two types of nanoparticles, MSN-PEG/TA (2:1) and MSN-PEG/TA (1:2), were used in this study. The details of the synthesis were described in the reports by Chen et al. [24]. Briefly, CTAB was used as a template, and then TEOS and RBITC were conjugated to synthesize red fluorescence MSNs by the sol–gel method. Subsequently, PEG-silane and TA-silane were added at a 2:1 and 1:2 ratio to obtain MSN-PEG/TA (2:1) and MSN-PEG/TA (1:2), respectively. Then, the particle suspension underwent an aging process followed by hydrothermal treatment for two days at 70 and 90 °C. A scanning electron microscope (SEM) image was taken by a JSM-75FCT (JEOL, Tokyo, Japan). A transmission electron microscope (TEM) image was taken by a JEM-2200FS (JEOL, Tokyo, Japan). The ζ-potential values of the MSNs were reported by Chen et al. in their previous work [24].

### 2.3. Cells and Media

Human cancer cell lines, namely OVCAR8-GFP ovarian cancer, A549 lung cancer, U87 glioblastoma, FaDu head and neck cancer, OS-46B’ (NCC-OS1-X2-C1) osteosarcoma [11] and OS-157 (NCC-ESOS1-C1) extraskeletal osteosarcoma [12], were cultured. OVCAR8 and 4T1 mouse breast cancer cells were provided by Carlotta Glackin (City of Hope Cancer Center). OS-46B’ and OS-157 were established and provided by the National Cancer Center of Japan. A549 was purchased from KAC Co., Ltd. (Kyoto, Japan). U87 and FaDu were purchased from ATCC (Manassas, VA, USA). OVCAR8 was seeded and maintained in RPMI1640 medium. A549, U87, OS-46B’ and OS-157 were maintained in DMEM medium. FaDu was maintained in EMEM medium. All these media were supplemented with 10% FBS (Fetal Bovine Serum, Thermo Fisher Scientific, Waltham, MA, USA) and 1% penicillin/streptomycin (Nacalai Tesque, Kyoto, Japan). As mouse cancer cell lines, CT26 colon cancer and 4T1 breast cancer were cultured. CT26 was provided by Minoru Suzuki (Kyoto Univ.). Both cell lines were seeded and maintained in RPMI1640 medium supplemented with 10% FBS and 1% penicillin/streptomycin.

### 2.4. Mouse Model

Ten-week-old female BALB/c mice received a transplantation of 8 × 10^5^ cells of CT26 mouse colon cancer subcutaneously in the right hind leg. Mice were acclimated in a controlled environment for over 1 week to allow enough of a tumor to form for experiments. At 9 days post-transplantation, tumor size was checked, and MSNs were injected into mice via the tail vein at 5 mg/mouse.

### 2.5. CAM Model

Fertilized white chicken eggs were purchased from Japan Layer (Gifu, Japan). The eggs were incubated at 37.5 °C with 65% humidity and automatically rotated once every hour. After preincubation for 10 days, 2 × 10^6^ cancer cells were transplanted onto the CAM. To transplant the cells, a 1.5 cm^2^ window was opened on the eggshell with a grinder. Then, the sterile Teflon ring (Tokyo Garasu Kikai Co., Ltd., Tokyo, Japan) was placed at a Y-shaped blood vessel branch. The cancer cells were seeded in the Teflon ring. After transplantation, the windows were sealed with Tegaderm film (3M Japan, Tokyo, Japan) to prevent dehydration. MSNs were administered at a dose of 1 mg/egg on day 9 post-transplantation. For the injection, new small windows were opened.

### 2.6. Biodistribution Study

The tumors and organs were harvested 3 h or 1 day after the injection of MSNs in the mouse and CAM models. Samples were prepared in sets of *n* = 3–4 per group. In the mouse model, the tumors and organs such as the liver, kidney and lung were observed by fluorescence microscopy, and the RBITC-labeled MSNs were detected. In the CAM model, the heart, spleen, stomach, intestine and brain were additionally observed in the same manner. The collected samples were fixed with 4% paraformaldehyde phosphate buffer solution (PFA) at 4 °C overnight. Then, the samples were washed with ice-cold PBS and treated with 20% sucrose solution overnight at 4 °C. Frozen sections were sliced at a thickness of 30 µm by a cryomicrotome. Nuclei were stained with Hoechst dye diluted 1:500 in PBS, and the thin sections were observed using a confocal laser scanning microscope (Nikon First-Scan Confocal Microscope A1R, Nikon Corporation, Tokyo, Japan) equipped with a 10× objective lens (CFI Plan Apo 10, Nikon Corporation, Tokyo, Japan). The excitation and emission wavelengths for Hoechst were 405 nm and 425–475 nm, respectively.

### 2.7. Tissue Ashing and Inductively Coupled Plasma Optical Emission Spectrometry (ICP-OES)

The tumors, blood and organs were weighed after harvest. Samples were prepared in sets of *n* = 3–4 per group. These samples were digested in lysis buffer consisting of 60% perchloric acid and 30% hydrogen peroxide (1:2) for 12 h at 75 °C. The ashing solution was filtered with a 0.22 µm membrane filter if residues were present. The final volume of these solutions was adjusted to 10 mL with sterile water, and the Si content was quantified using a Shimadzu ICPE-9820 (Kyoto, Japan).

### 2.8. Histological Analysis of Tumors in the Mouse and CAM Models

After the transplantation of cancer cells, tumors developed either on the hind legs of the mice or on the CAMs. Samples were prepared in sets of *n* = 1–6 per group. Tumors were harvested at different time points between day 3 (ED11) and day 10 (ED20) after transplantation. The collected tumors were fixed with 10% formalin neutral buffer solution for the mice and 4% PFA for the CAMs and stored at 4 °C overnight. After fixation, tissues were embedded in paraffin, and thin sections were sliced at a thickness of 3 µm. These sections were subjected to hematoxylin and eosin (H&E) staining. Both the paraffin embedding and H&E staining were performed by Kyoto Institute of Nutrition & Pathology, Inc. (Kyoto, Japan). The histological morphology was observed using a Keyence BZ-9000 or BZ-X810 (Osaka, Japan). While we performed only H&E staining in this study, this approach was considered a good indication based on our previous confirmation of the reflection of the original tumor including a fusion gene [10].

## 3. Results

### 3.1. CAM Models Established from Various Human Cancer Cell Lines

**Figure 1:**
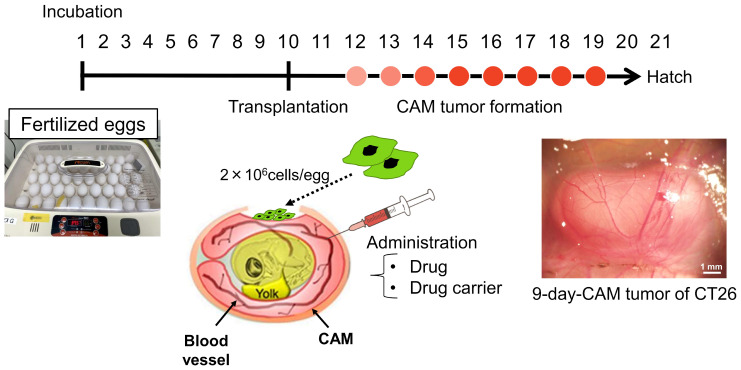
Experimental flow of the CAM assay and a representative CT26 CAM tumor 9 days after transplantation. The CAM tumor gradually developed after transplantation. The scale bar represents 1 mm.

**Figure 2:**
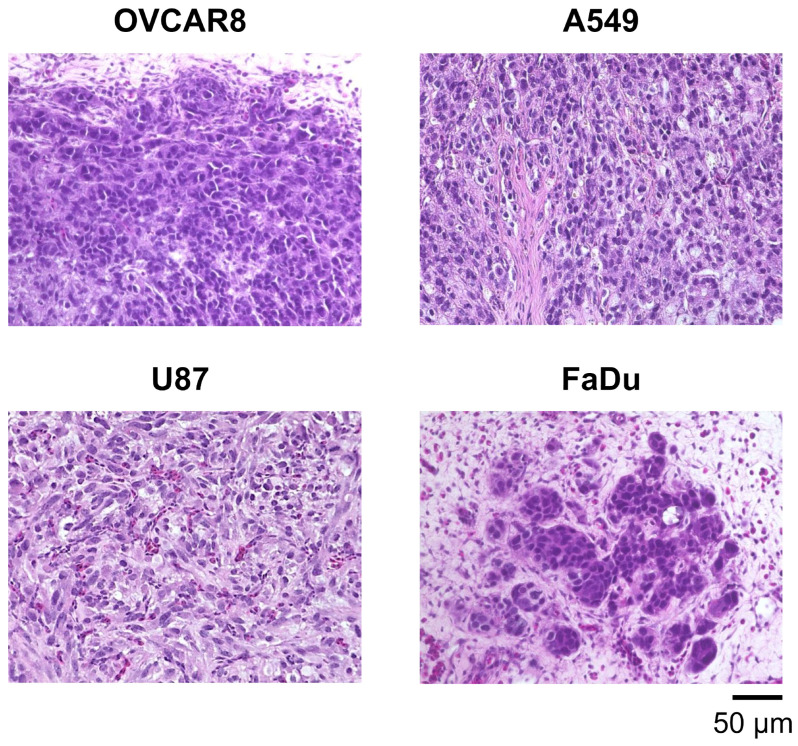
Histological analysis of CAM tumors using H&E staining. CAM tumors were observed after the transplantation of human cancer cells, ovarian cancer (OVCAR8), lung cancer (A549), glioblastoma (U87) and head and neck cancer (FaDu).

The establishment of various types of CAM tumor models can facilitate cancer research in areas such as molecular mechanism analysis, drug screening, tumor microenvironment studies and precision medicine. Therefore, we examined several types of cancer cell lines, including patient-derived and mouse cancer cell lines. These cell lines were transplanted at 2 × 10^6^ cells per egg onto CAMs between embryonic day (ED) 8 and ED10, and cancer cell growth within the CAM tumors was observed using H&E staining (Figure 1, Table 1). All CAM tumors gradually grew after transplantation, and the cancer cells within the CAM tumor increased daily. Human cancer cells, OVCAR8, A549, U87 and FaDu, exhibited different patterns of growth within the CAM tumors (Figure 2).

### 3.2. CAM Models Established from Patient-Derived Osteosarcoma Cell Lines

**Figure 3:**
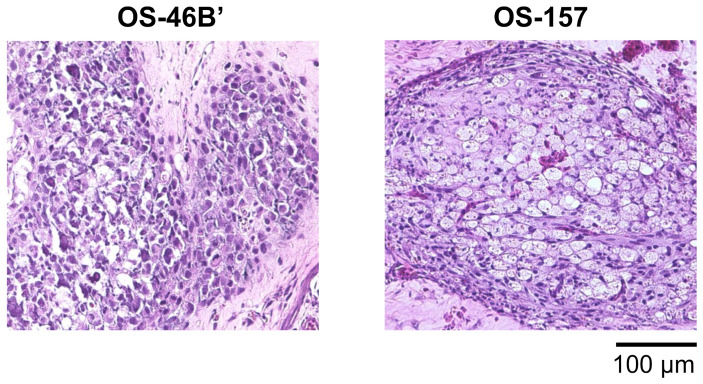
Histological analysis of CAM tumors using H&E staining. Establishment of CAM tumors using patient-derived osteosarcoma (OS-46B’) and extraskeletal osteosarcoma (OS-157).

Also, we successfully established CAM tumors using patient-derived cells, OS-46B’ and OS-157 (Figure 3). Unlike OVCAR8 and A549, these tumors grew in a two-dimensional, planar manner on CAMs. These findings suggest that CAM tumors have the potential to mimic and reflect the distinct characteristics of each cancer type. The growth pattern within the CAM tumors was evaluated based on visual observation of the distribution ratio per unit area of the CAM section as follows: − (0%), + (<50%), and ++ (≥50%) (Table 1). This visual classification method was employed because the proliferation patterns of cancer cells in CAM tumors were clearly distinguishable as either partial or widespread. The proliferation of U87 and CD-292 cancer cells increased rapidly and spread throughout the CAM tumor. The morphology of CD-292 was reported in our previous work [10].

### 3.3. CAM Models Established from Mouse Cell Lines

**Figure 4:**
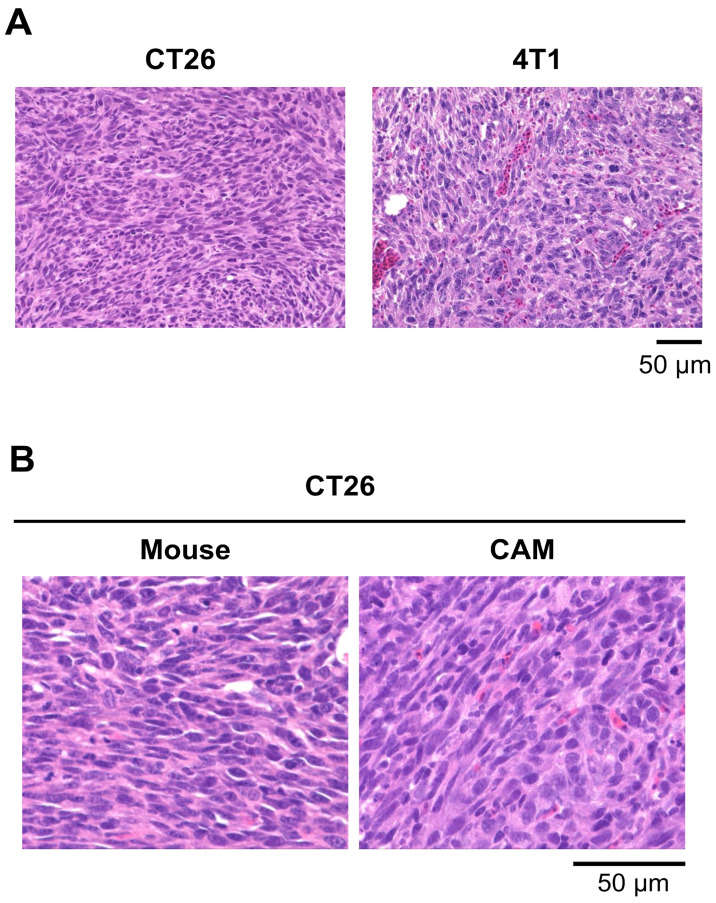
Histological analysis of CAM tumors using mouse cancer cells by H&E staining. (**A**) Morphology of CAM tumors using CT26 and 4T1 cells. In the 4T1 tumor, numerous nucleated red blood cells, characteristic of avian species, were observed infiltrating the tumor tissue. (**B**) Morphological similarity of CT26 tumors between the mouse and CAM models.

We also established CAM models using mouse cancer cell lines. Mouse cancer cells, CT26 and 4T1, were transplanted onto the CAMs in the same manner as human cancer cells. Both CAM tumors developed, and Figure 1 presents a representative CT26 CAM tumor on day 9 post-transplantation. H&E staining of tissue sections revealed that the cancer cells proliferated and spread throughout the CAM tumor (Figure 4A). During the development of 4T1 CAM tumors, frequent bleeding was observed, which may suggest that the 4T1 tumors rapidly induced vascularization. Thus, various differences can be observed during the tumor growth process, depending on the individual cancer type. A comparison of CT26 tumors in the CAM and mouse models revealed similar morphological characteristics in histological analysis by H&E staining (Figure 4B). In the mouse model, CT26 cells were implanted subcutaneously on the leg.

### 3.4. Two Types of Positively Charged, Small-Size, Highly Dispersive MSNs (sdMSNs)

**Figure 5:**
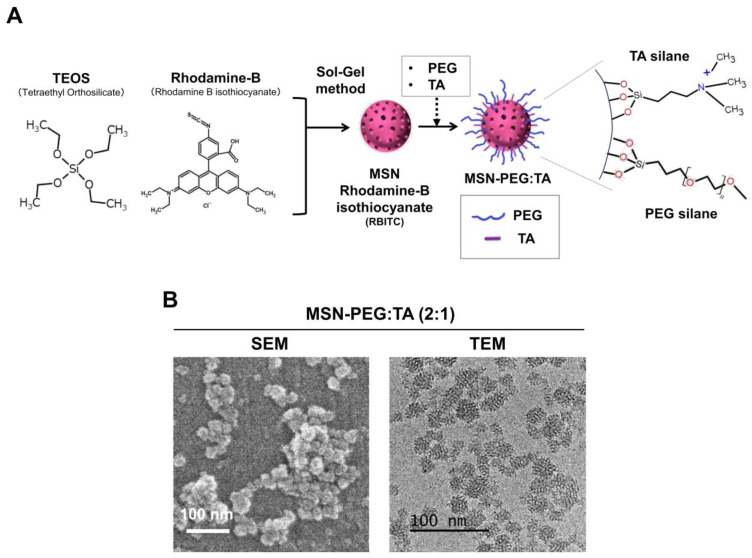
Synthesis and characteristics of MSNs. (**A**) Synthesis method and modification of surface charge. (**B**) Characteristic images of MSN-PEG/TA (2:1) obtained using a scanning electron microscope (SEM) and transmission electron microscope (TEM).

The establishment of the CT26-cell-line-derived CAM model and mouse xenografts provided an opportunity to compare the two animal models, CAM and mouse. To do this, we decided to use two types of recently developed small-size, highly dispersive MSNs (sdMSNs) [24], MSN-PEG/TA (2:1) and MSN-PEG/TA (1:2). These nanoparticles were synthesized via a sol–gel method using CTAB as a template. TEOS was used to form the main silica framework of sdMSNs, and RBITC was introduced to label the particles with red fluorescence. The particle size was controlled by adjusting the pH of the reaction solution during synthesis. Subsequently, PEG-silane and TA-silane were added at ratios of 2:1 and 1:2, respectively, to prepare MSN-PEG/TA (2:1) and MSN-PEG/TA (1:2). These formulations allowed the control of surface charge. The former exhibited a weakly positive charge, while the latter showed a stronger positive charge (Figure 5A). The ζ-potential of MSN-PEG/TA (2:1) was +4.0 mV, while that of MSN-PEG/TA (1:2) was +21.0 mV [24]. In addition to surface charge, the high dispersibility of these MSNs is also a key characteristic that could be affected by various factors during synthesis. Among these, the hydrothermal treatment or aging process is particularly significant when using PEG-silane as a surface modifier [28]. These processes enhance the condensation of the surface silica network, a feature that is unique to silica nanoparticles due to sol–gel-based chemistry [29]. While MSNs can be modified using conventional linker–PEG strategies—commonly employed for polymers, liposomes, and gold nanoparticles—they also possess the unique ability to incorporate PEG via silane chemistry. This method enables PEG to directly bond with the silica network, forming stable Si–O linkages that enhance nanoparticle stability and avoid aggregation. The size and morphology of MSN-PEG/TA (2:1) were confirmed using a scanning electron microscope (SEM) and transmission electron microscope (TEM) (Figure 5B). The size was approximately 25 nm. Similar results were obtained for MSN-PEG/TA (1:2).

### 3.5. Characterization of Tumor Accumulation of sdMSNs Using the CAM and Mouse Models

Recently, Chen et al. demonstrated that positively charged PEGylated MSNs administered intravenously to xenografted mice (4T1/Balbc) [24] preferentially accumulated in tumors within 24 h; Weakly positive MSN-PEG/TA (2:1) accumulated more preferentially in tumors compared with strongly positive MSN-PEG/TA (1:2). Furthermore, MSN-PEG/TA (2:1) showed minimal localization to other organs, such as the liver, lung, kidney, heart and spleen. This result highlights the importance of optimizing nanoparticle size, PEGylation and surface charge, as excess positive charge may inhibit selective tumor targeting. We first characterized their capabilities in the mouse model using CT26 cells.

#### 3.5.1. Biodistribution of sdMSNs in CT26-Bearing Mouse Model

**Figure 6:**
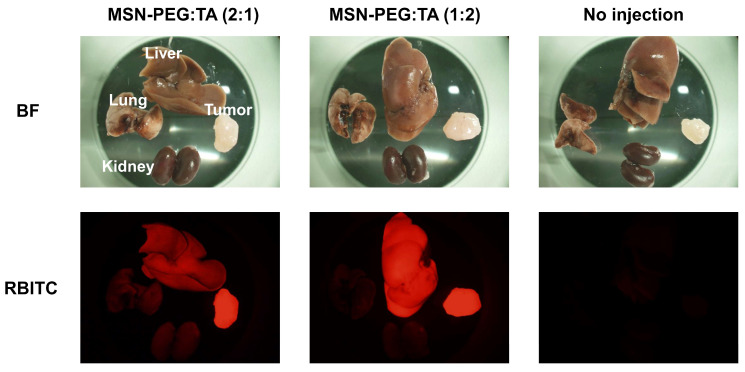
Biodistribution of MSNs in CT26-bearing mouse model. The mice were intravenously injected with sdMSNs at a dose of 5 mg/mouse. Magnification; 35×.

Both types of MSNs were intravenously injected into CT26-bearing mice at a dose of 5 mg/mouse. After 24 h, the tumor and major organs (liver, kidney and lung) were harvested and analyzed using fluorescence microscopy. Red fluorescence from rhodamine B-labeled MSNs indicated successful tumor accumulation of both MSN-PEG/TA (2:1) and MSN-PEG/TA (1:2) (Figure 6). However, MSN-PEG/TA (2:1) exhibited a higher tumor-targeting ability than MSN-PEG/TA (1:2). These findings further support the tumor accumulation results observed in the 4T1-bearing mouse model [24].

#### 3.5.2. Localization of MSN-PEG/TA (2:1) in Mouse Tumors and Organs

**Figure 7:**
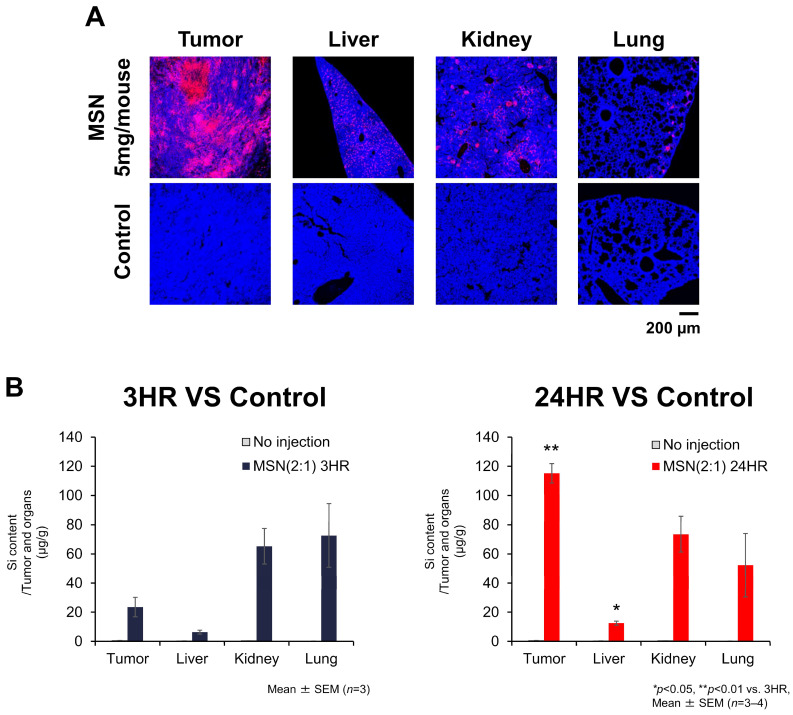
Localization of MSN-PEG/TA (2:1) in tumors and organs in the mouse model. (**A**) Confocal microscopic images of tumors and organs. The upper panel shows images of samples injected with 5 mg of MSNs per mouse. The lower panel shows no injection samples. Red color indicates rhodamine B-labeled MSNs, and blue color indicates nuclei. (**B**) The silicon content in tumor, liver, kidney and lung in CT26-bearing mice was quantified using ICP-OES. The samples were harvested 3 h and 24 h after MSN injection.

To further examine the localization of MSN-PEG/TA (2:1) in tumors and organs in the mouse model, the tumors and organs were harvested, and thin sections were prepared. As previously mentioned, MSN-PEG/TA (2:1) exhibited excellent tumor selectivity, with the red fluorescence intensity being extremely high under fluorescence microscopy. The confocal images in Figure 7A show that RBITC-labeled MSNs spread throughout the tumor. In other organs such as the liver, kidney and lung, the red fluorescence was significantly lower than that in the tumor. These findings are consistent with the results of the biodistribution analysis in Figure 6.

To quantitate the accumulation of MSN-PEG/TA (2:1) in the CAM tumors as well as in major organs, amounts of silicon (Si) were detected by ICP-OES. Tumors, organs and blood were harvested at 3 h and 24 h after the injection of MSNs (Figure 7B). Since MSN-PEG/TA is predominantly composed of Si, the Si content was quantified by ICP. ICP analysis revealed that the Si content in tumor tissue reached 115 µg/g tissue at 24 h after the injection of MSN-PEG/TA (2:1) (mean ± SEM: 115.2 ± 15.1 µg/g tissue, *n* = 4). This accumulation level was notably higher than that in the liver (mean ± SEM: 12.5 ± 0.9 µg/g tissue, *n* = 4), a representative clearance organ. This tumor selectivity is remarkable for systemically administered nanoparticles. It is also consistent with the strong fluorescence signal observed in Figure 7A. Compared to 3 h, the Si content per gram of tumor tissue increased approximately 5-fold at 24 h, while that in the liver doubled. In contrast, no significant changes were observed in the blood and other organs (Figure 7B).

#### 3.5.3. Biodistribution of MSNs in the CAM Model and Its Similarity to the Mouse Model

**Figure 8:**
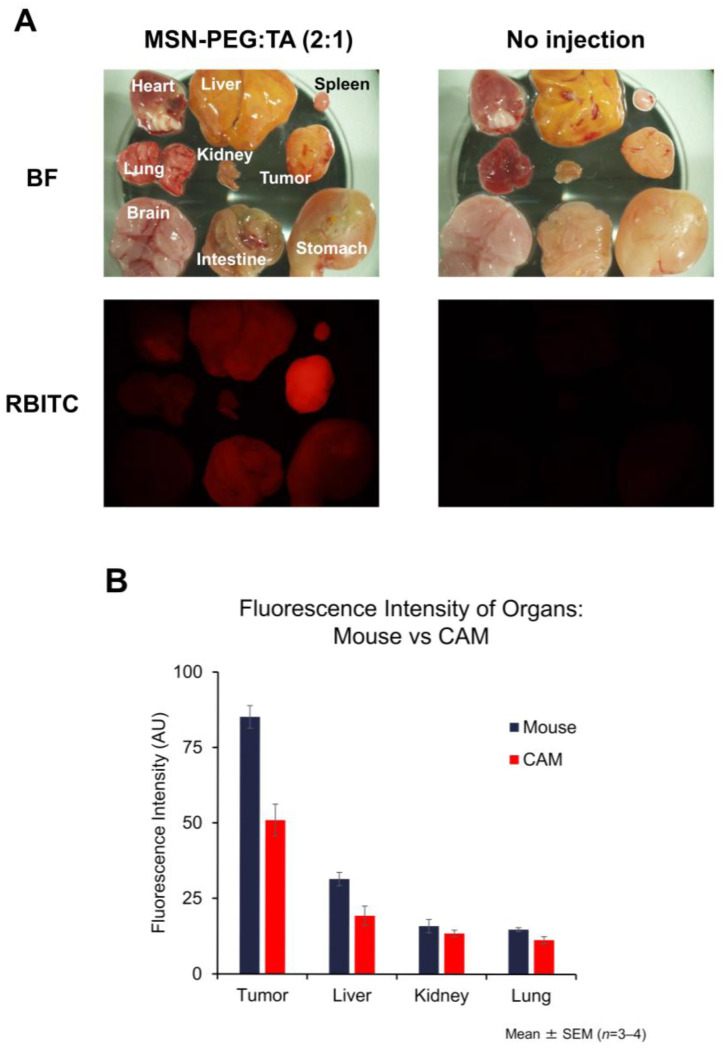
Biodistribution of MSNs in the CT26-bearing CAM model. The eggs were administered MSN-PEG/TA (2:1) at a dose of 1mg/egg. (**A**) Tumor and organs injected with MSN were harvested at 24 hours post-injection. Magnification; 35×, (**B**) Fluorescence intensity of tumors and organs in the CAM model compared to that in the mouse model. Both tumors were established using CT26 cells.

Since we were convinced by the excellent tumor accumulation capability of MSN-PEG/TA (2:1) in the CT26-bearing mouse model, we then examined the accumulation of MSNs in the CAM tumors. CT26 cells (2 × 10^6^ cells) were transplanted onto CAMs of 10-day-old embryos. CAM tumors gradually formed after transplantation. On day 9 post-transplantation (ED19), MSN-PEG/TA (2:1) was intravenously injected into the eggs. Tumors and organs were harvested 1 day post-injection, and the biodistribution was analyzed using fluorescence microscopy (Figure 8A). The observed biodistribution patterns were similar to those in the mouse model (Figure 6), whereas no fluorescence of MSNs could be seen in the no-injection control. For further characterization of tumor accumulation, the red fluorescence intensity in both the CAM and the mouse models was quantified using ImageJ software (version 1.54p, NIH, Bethesda, MD, USA) (Figure 8B). Quantitative analysis revealed a consistent trend and demonstrated a strong correlation with the organ distribution, including tumors, between the two models.

## 4. Discussion

In this study, we established CAM models using various cancer cell lines. First, we transplanted human cell lines, OVCAR8 (ovarian cancer), A549 (lung cancer), U87 (brain tumor) and FaDu (head and neck cancer), onto chicken egg CAMs, resulting in the formation of CAM tumors in approximately 5 days. The presence of cancer cells in each CAM tumor was confirmed using H&E staining. Cancer cells within the CAM tumors increased rapidly. With U87 cells, in particular, they proliferated rapidly and spread throughout the CAM tumors.

We transplanted two types of patient-derived osteosarcoma cell lines, OS-46B’ and OS-157, which were provided by the National Cancer Center of Japan [11,12]. At first glance, these CAM tumors appeared underdeveloped. However, histological observation using H&E staining clearly revealed the proliferation of cancer cells. Extraskeletal osteosarcoma (ESOS) is a rare cancer with poor prognosis and limited treatment options, making it difficult to study. In addition to our previous success in establishing patient-derived *CIC-DUX4* sarcoma cells and confirming the retention of the *CIC*-*DUX4* fusion gene [10], we have significantly expanded the opportunity to use CAMs for the characterization of patient-derived cancer samples.

The establishment of CAM models from mouse cell lines enabled us to characterize the tumor accumulation capability of the recently developed small-size, highly dispersive mesoporous silica nanoparticles (sdMSNs) in both CAM and mouse models. Chen et al. first confirmed and extended the previous report of the excellent tumor accumulation capability of small-size, highly dispersive MSNs in a 4T1 mouse model [24]. In our study, using a CT26 model, the tumor localization of sdMSNs was observed by 3 h after the intravenous injection of sdMSNs. This tumor accumulation increased by 24 h, and at this point, preferential tumor accumulation was observed. A comparison of the two types of sdMSNs with different charges (PEG/TA (2:1) and MSN-PEG/TA (1:2)) in the CT26-bearing mouse model showed that the weakly positively charge MSN-PEG-TA (2:1) exhibited excellent tumor accumulation compared with the more positively charged MSN-PEG/TA (1:2). Confocal analysis of tumors and organs (liver, kidney and lung) injected with MSN-PEG/TA (2:1) was consistent with the results of fluorescence microscopy. These results demonstrate the tumor-selective localization of sdMSNs in two different tumor mouse models. The preferential accumulation of MSNs in the CAM tumors was further confirmed by examining the Si content by ICP. Compared with the samples at 3 h, the Si content increased 5-fold in the tumor and 2-fold in the liver at 24 h, while no significant difference was observed in other organs and blood. Interestingly, the Si content in blood was almost the same between 3 h and 24 h, pointing to a prolonged circulation of these nanoparticles. The characterization using two animal models, the CAM and mouse models, revealed similar tumor-targeting and biodistribution profiles of the sdMSNs. This result confirms the validity of the CAM model as a simple and versatile in vivo system for evaluating tumor accumulation. In addition, the similarity in biodistribution between the CAM and mouse models could broaden the choice of preclinical animal models in further tumor accumulation studies. Due to the rapid tumor formation, low cost and technical simplicity of the CAM model (Figure 1), it could be used as a practical and efficient screening platform before mouse experiments. This would help accelerate the development and evaluation of nano-based drug carriers.

One of the promises of nanomedicine is to achieve tumor targeting of anticancer drugs that could decrease side effects associated with small-molecule drugs. This promise was based on the idea that nanoparticles can accumulate in the tumor by taking advantage of leaky vasculature in the tumor (Enhanced Permeability and Retention, EPR effect) [30]. To achieve this, it is necessary to have a material that has prolonged blood circulation so that there will be enough time for tumor accumulation to take place. In addition, nanoparticles have to be well dispersed and not be captured in the liver. The small-size, highly dispersed MSNs described in this study fit these criteria. However, further work is needed to characterize their tumor accumulation in CAMs and in mice.

## 5. Conclusions

In this study, we successfully broadened the applicability of the CAM model by employing a variety of cancer cells, including patient-derived cell lines. Furthermore, our findings demonstrated that recently developed sdMSNs exhibit strong tumor accumulation in both CAM and mouse models. These findings expand our previous work using *CIC-DUX4* sarcoma, further supporting the applicability of the CAM model to patient-derived cancer modeling. This model also serves as a valuable tool for evaluating tumor-targeting studies.

## Figures and Tables

**Table 1:** Transplanted cancer types and assessment of tumor proliferation using H&E staining. The growth pattern within the CAM tumor was evaluated based on visual observation of the distribution of cancer cells per area of the CAM section as follows: − (0%), + (< 50%), and ++ (≥ 50%).

Origin	Cancer Type	Cell Line	Collection Date (Days Post-Transplantation)	Transplantation Date (ED: Embryonic Days)	Tumor Proliferation (+/−)
Established human cell line	Glioblastoma	U87	3–7, 9, 10	ED9–10	++
	Head and neck cancer	FaDu	7, 9	ED8	+
	Lung cancer	A549	3, 6, 10	ED10	+
	Ovarian cancer	OVCAR8	3–7, 9, 10	ED8–10	+
Patient-derived cell line	Osteosarcoma	OS–46B’	4, 6, 8	ED8	+
	Extraskeletal osteosarcoma	OS–157	7, 10	ED10	+
	CIC-DUX4 sarcoma	CD–292	3, 5, 7, 10	ED8	++
		CD–89A, CD–89C	7, 10	ED8	+
Mouse cell line	Breast cancer	4T1	9	ED10	++
	Colon cancer	CT26	3, 7, 8	ED10	++
Only CAM		−	3, 6, 9	ED10	−

## Data Availability

The data presented in this study are available in the article and the Supplementary Materials.

## Supplementary Materials

The following supporting information can be downloaded at https://www.mdpi.com/article/10.3390/cells14100734/s1: Figure S1: Appearance of various CAM tumors.
