# Supplementary material for: Accumulation of Small-Size, Highly Dispersive Mesoporous Silica Nanoparticles in a Tumor in Both Chorioallantoic Membrane and Mouse Models
Source: Cells. 2025 May 17;14(10):734. doi: 10.3390/cells14100734 (PMC12110715; doi:10.3390/cells14100734)
Supplement: Supplementary file 1 [file cells-14-00734-s001.zip › cells-3636425-supplementary.pdf]

cells

MDPI

# Supplementary Materials: Accumulation of small-size, highly-dispersive mesoporous silica nanoparticles (MSN) in the tumor in both CAM and mouse models

**Aoi Komatsu [1], Yuya Higashi [1], Cong-Kai Lin [2,3], Yi-Ping Chen [2,3], Si-Han Wu [2,3], Minoru Suzuki [4], Kotaro Matsumoto [1,*] and Fuyuhiko Tamanoi [1,*]**

1 Institute for Integrated Cell-Material Sciences, Institute for Advanced Study, Kyoto University, Kyoto 606-8501, Japan; komatsu.aoi.6z@kyoto-u.ac.jp (A.K.), higashi.yuya.5n@kyoto-u.ac.jp (Y.H.)
2 Graduate Institute of Nanomedicine and Medical Engineering, Taipei Medical University, Taipei 11031, Taiwan; qrq123456789@gmail.com (C.-K.L.), haychen@tmu.edu.tw (Y.-P.C), smilehanwu@tmu.edu.tw (S.-H.W.)
3 International Ph.D. Program in Biomedical Engineering, Taipei Medical University, Taipei 11031, Taiwan
4 Institute for Integrated Radiation and Nuclear Science, Kyoto University; suzu-ki.minoru.3x@kyoto-u.ac.jp (M.S.)
* Correspondence: matsumoto.kotaro.5r@kyoto-u.ac.jp (K.M.), tamanoi.fuyuhiko.2c@kyoto-u.ac.jp (F.T.); Tel.: +81-75-753-9856

## 1. Appearance of various CAM tumors

In this study, we transplanted various types of cancer cell lines, OVCAR8 ovarian cancer, A549 lung cancer, U87 glioblastoma, FaDu head and neck cancer and patient-derived cancer cell lines such as OS-46B' osteosarcoma and OS-157 extraskeletal osteosarcoma. Bright-field images of representative CAM tumors formed by OVCAR8, U87, OS-46B' and OS-157 are shown in Supplementary Figure S1.

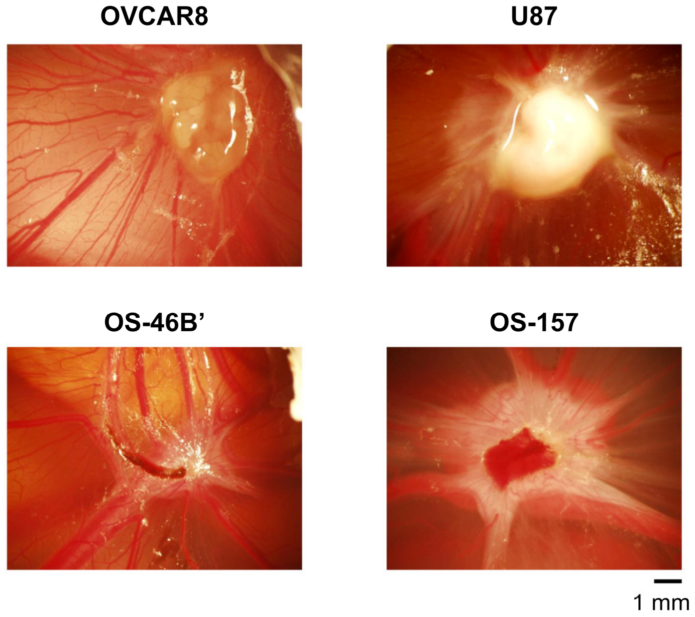


Figure S1. Bright-field images of CAM tumors using OVCAR8, U87, OS-46B' and OS-157

**Disclaimer/Publisher's Note:** The statements, opinions and data contained in all publications are solely those of the individual author(s) and contributor(s) and not of MDPI and/or the editor(s). MDPI and/or the editor(s) disclaim responsibility for any injury to people or property resulting from any ideas, methods, instructions or products referred to in the content.
